# Interferon-γ Produced by EBV-Positive Neoplastic NK-Cells Induces Differentiation into Macrophages and Procoagulant Activity of Monocytes, Which Leads to HLH

**DOI:** 10.3390/cancers13205097

**Published:** 2021-10-12

**Authors:** Mayumi Yoshimori, Miwako Nishio, Ayaka Ohashi, Megumi Tateishi, Ayaka Mimura, Naomi Wada, Minori Saito, Norio Shimizu, Ken-Ichi Imadome, Ayako Arai

**Affiliations:** 1 Department of Hematological Therapeutics, Graduate School of Medical and Dental Sciences, Tokyo Medical and Dental University (TMDU), Tokyo 113-8519, Japan; yoshlmg@tmd.ac.jp; 2 Department of Laboratory Molecular Genetics of Hematology, Graduate School of Medical and Dental Sciences, Tokyo Medical and Dental University (TMDU), Tokyo 113-8519, Japan; mnishio.lmg@tmd.ac.jp (M.N.); ayaka.ohashi@marianna-u.ac.jp (A.O.); ma190062@tmd.ac.jp (M.T.); ma200079@tmd.ac.jp (A.M.); ma210045@tmd.ac.jp (M.S.); 3 Department of Frontier Medicine, Institute of Medical Science, St. Marianna University School of Medicine, Kawasaki 216-8511, Japan; 4 Department of Advanced Medicine for Infections, National Center for Child Health and Development (NCCHD), Tokyo 157-8535, Japan; wada-n@ncchd.go.jp (N.W.); imadome-k@ncchd.go.jp (K.-I.I.); 5 Center for Stem Cell and Regenerative Medicine, Tokyo Medical and Dental University (TMDU), Tokyo 113-8519, Japan; nshivir@tmd.ac.jp; 6 Division of Hematology and Oncology, Department of Internal Medicine, St. Marianna University School of Medicine, Kawasaki 216-8511, Japan

**Keywords:** EBV-positive T- or NK-cell neoplasms, hemophagocytic lymphohistiocytosis, disseminated intravascular coagulation, interferon-γ, macrophage, differentiation, procoagulant activity

## Abstract

**Simple Summary:**

Epstein–Barr virus (EBV), a common virus all over the world, infects not only B-cells but also T- and NK-cells. Once infected with EBV, human beings remain infected for life, and EBV renders infected B-cells immortal. EBV-positive NK-cell neoplasms, such as extranodal NK/T-cell lymphoma of nasal type, aggressive NK-cell leukemia, and chronic active EBV infection, are relatively rare but lethal disorders. They show systemic inflammation and progress to hemophagocytic lymphohistiocytosis (HLH), a life-threatening state of immune hyperactivation. The suppression and prevention of HLH are important to treat the neoplasms. Revealing the mechanism will pave a new path for treatment. We show herein that IFN-γ produced by EBV-positive neoplastic NK-cell is responsible for inducing the differentiation and the activation of M1-like macrophages. Suppressing IFN-γ may regulate HLH in EBV-positive NK-cell neoplasms.

**Abstract:**

Epstein–Barr virus (EBV)-positive T- or NK-cell neoplasms show progressive systemic inflammation and abnormal blood coagulation causing hemophagocytic lymphohistiocytosis (HLH). It was reported that inflammatory cytokines were produced and secreted by EBV-positive neoplastic T- or NK-cells. These cytokines can induce the differentiation of monocytes into macrophages leading to HLH. To clarify which products of EBV-positive neoplastic T- or NK-cells have effects on monocytes, we performed a co-culture assay of monocytes with the supernatants of EBV-positive T- or NK-cell lines. The expression of differentiation markers, the phagocytosis ability, and the mRNA expression of the inflammatory cytokines of THP-1, a monocytic cell line, clearly increased after culturing with the supernatants from EBV-NK-cell lines. Co-culturing with the supernatants promoted the expression of CD80 and CD206 as well as M1 and M2 macrophage markers in human monocytes. Co-culturing with the supernatants of EBV-NK-cell lines significantly enhanced the procoagulant activity and the tissue factor expression of monocytes. Interferon (IFN)-γ was elevated extremely not only in the supernatant of EBV-NK-cell lines but also in the plasma of EBV-positive NK-cell neoplasms patients accompanying HLH. Finally, we confirmed that IFN-γ directly enhanced the differentiation into M1-like macrophages and the procoagulant activity of monocytes. Our findings suggest that IFN-γ may potentially serve as a therapeutic target to regulate HLH in EBV-positive NK-cell neoplasms.

## 1. Introduction

Epstein–Barr virus (EBV) belongs to the human herpes virus family and mainly targets B lymphocytes. Although it is less common, EBV also infects T cells and natural killer (NK) cells. This infection is detected in EBV-positive T- or NK-cell neoplasms, such as extranodal nasal NK/T-cell lymphoma, aggressive NK-cell leukemia (ANKL), and chronic active EBV infection (CAEBV). These neoplasms are rare and have geographical preference to East Asia and South America. They show systemic inflammation and progress to hemophagocytic lymphohistiocytosis (HLH).

HLH is characterized as a severe inflammation caused by the uncontrolled activation of lymphocytes and macrophages [1]. Most adult-onset HLH is secondary HLH, which develops during the course of immune disorders, infectious diseases, or neoplasms [2,3]. Among the underlying disorders, EBV-associated diseases including EBV-positive lymphomas are the most common diseases making up 29% of the total [4]. Once HLH develops, cytopenia and multi-organ failure caused by disseminated intravascular coagulation (DIC) progress rapidly, and the condition becomes lethal [2,5]. Since HLH is one of the main causes of death in EBV-positive T- or NK-cell neoplasms, the regulation of HLH is crucial in treating the disease. Resolving the pathogenic mechanisms and the establishment of effective treatments are urgent issues.

Inflammatory cytokines, such as tumor necrosis factor (TNF)-α, interleukin-6 (IL-6), and interferon (IFN)-γ, whose concentrations in the plasma are elevated in HLH, induce the differentiation of monocytes to macrophages [6,7]. In addition, the expression of tissue factor (TF), which plays a pivotal role in promoting coagulation to cause DIC, increases inducibly in monocytes stimulated by the inflammatory cytokines of TNF-α [8], IL-6 [9], and IFN-γ [10]. These findings suggest that the inflammatory cytokines are the key factors in developing HLH.

There are reports on their elevated plasma concentrations in EBV-positive T- or NK-cell neoplasms [11,12,13]. There are also reports on the enhanced expression of mRNA of *TNF-α* and *IFN-γ* in EBV-positive T- or NK-cells. Thus, we hypothesized that some cytokines produced by EBV-positive neoplastic T- or NK-cells may induce blood coagulation and macrophage activation resulting in DIC and HLH. To prove our hypothesis and to establish an effective treatment for HLH in EBV-positive T- or NK-cell neoplasms, we investigated the products secreted from EBV-positive T- or NK-neoplastic cells. We aimed to determine their effects on macrophages and the responsible factors for the effects.

## 2. Materials & Methods

### 2.1. Cells and Reagents

SNT8 was derived from T cell type of Nasal T/NK cell lymphoma. SNK6 was derived from the NK cell type of Nasal T/NK cell lymphoma. SNT15 and SNT16 were derived from T cell type of CAEBV. SNK1 and SNK10 were derived from NK cell type of CAEBV [14]. They were cultured in Artemis medium-2 (Nihon Techno Service, Ibaragi, Japan) [14]. A monocytic leukemia cell line, THP-1, was obtained from Health Science Research Resources Bank (Osaka, Japan). Human primary monocytes were obtained from healthy donors using Pan Monocyte Isolation Kit (Miltenyi Biotec, Bergisch Gladbach, Germany). NK-cells were also obtained from healthy donors using CD56 magnetic beads (Miltenyi Biotec).

They were both isolated from their peripheral blood mononuclear cells (PBMC) using lymphoprep (Abbott Diagnostics Technologies AS, Chicago, IL, USA). They were cultured in RPMI-1640 (Wako Pure Chemical Industries, Osaka, Japan) containing 10% fetal bovine serum (Sigma-Aldrich, St. Louis, MO, USA). Recombinant human TNF-α (300-01A), IL-6 (200-06) and IFN-γ (300-02-1) were purchased from PeproTech (East Windsor, NJ, USA). Phorbol 12-myristate 13-acetate (PMA) was purchased from Sigma-Aldrich.

### 2.2. Stimulation of Target Cells with the Supernatants of EBV-Positive Cell Lines

The supernatants of EBV-positive cell lines were obtained from exponentially growing EBV-positive cell lines. These supernatants were added to the culture media of the target cells. The same amount of Artemis Medium-2 was added to the culture media as untreated controls. After incubating for indicated times, the cells were collected for assays.

### 2.3. Morphological Analysis

After the stimulation of target cells, they were observed under the microscope (IX73, Olympus, Tokyo, Japan).

### 2.4. Detection of Cell Surface Antigens by Flow Cytometry

After the stimulation, the target cells were analyzed by a FACSCalibur™ flow cytometer (Becton Dickinson and Company, Franklin Lakes, NJ, USA) with FITC-conjugated mouse anti-human CD11b (ab176541, abcam, Cambridge, UK), FITC-conjugated mouse anti-human CD80 (560926, BD Pharmingen, Franklin Lakes, NJ, USA), FITC-conjugated mouse anti-human CD206 (321103, Biolegend, San Diego, CA, USA), and monoclonal mouse anti-human TF IgG (ADG4509; American Diagnostics, Greenwich, CT, USA) with FITC-labeled anti-mouse IgG antibody (1030-02, SouthernBiotech, Birmingham, AL, USA). 

To confirm the enrichment of human monocytes, PBMC and human monocytes isolated by Pan Monocyte Isolation Kit were analyzed with FITC-conjugated mouse anti-human CD14 (555397, BD Pharmingen). The mean fluorescent intensity was normalized by isotype-matched control, IgG1 (Mouse)-FITC (A07795, BECKMAN COULTER, Fullerton, CA, USA) and expressed as the mean fluorescence intensity rate (MFIR). Phosphatidylserine (PS) was detected using ApoScreen™ (R) Annexin V antibody (10040-02, SouthernBiotech) in accordance to the manufacturer’s instructions.

### 2.5. Analysis of the Phagocytosis Ability

After stimulating THP-1 cells, FluoSpheres™ Polystyrene Microspheres (F13082, Invitrogen, Waltham, MA, USA) were added to the culture medium at 4 × 10^6^ beads/mL as manufacture’s protocol, and they were left to take up the beads for 4 h at 37 °C. After the bead uptake, cells were analyzed using a FACSCalibur™ flow cytometer. The rate of the positive fluorescence intensity was estimated as the phagocytosis ability.

### 2.6. qRT-PCR

RNA was extracted from the target cells with ISOGEN II (Nippon Gene, Tokyo, Japan) in accordance to the manufacturer’s protocol, and cDNA was generated using PrimeScript RT Master Mix (TAKARA, Shiga, Japan). qRT-PCR analysis was performed on a Light Cycler 480 (Roche, Basel, Switzerland) using TaqMan^®^ Gene Expression Assays (Applied Biosystems, Foster City, CA, USA). The expressions of IL-6 (Hs00985639_m1), IL-8 (Hs00174103_m1), TNF-α (Hs01113624_g1), and TF (Hs01076029_m1) were normalized to the expression of human GAPDH (4325792).

### 2.7. Procoagulant Activity (PCA) Assay

A portion of target cells (2 × 10^6^) was suspended in 50 μL phosphate-buffered saline (PBS) and added to 50 μL of healthy human plasma. After the incubation at 37 °C for 3 min, 50 μL of 25 mM calcium chloride was added, and the plasma recalcification time was measured using a semi-automatic coagulator (CA-50, Sysmex, Kobe, Japan) [15]. CA-50 detected changes in plasma optical density during blood coagulation. A shortened coagulation time indicated increased PCA.

### 2.8. Cell Surface PCA Blocking Assay

To investigate the effects of cell surface TF, the stimulated target cells were treated with either 2.5 μg/mL of monoclonal anti-TF antibody (ADG4509; American Diagnostics) or the same amount of irrelevant IgG in PBS for 60 min at 4 °C. After washing with PBS, cell surface PCA was assessed as described above.

To investigate the effects of cell surface PS exposure, the stimulated target cells were treated with 1 μg/mL of Annexin V antibody (10040-02, Southern Biotechnology, Boca Raton, FL, USA) in Annexin V binding buffer at 37 °C for 30 min. After the incubation, the cell surface PCA was assessed as described above.

### 2.9. Cytokine Multiplex Analysis

Cytokine multiplex analysis was performed using the Human Cytokine/Chemokine Panel 38 Plex (EMD Millipore) in accordance with the manufacturer’s instructions. The cytokine levels were determined using the Luminex 200 System (Luminex Corporation, Austin, TX, USA).

### 2.10. Statistical Analysis

We present the data as the mean ± standard deviation (SD). We processed the statistical analysis by two-tailed Student’s *t*-test using GraphPad Prism 5 (GraphPad, San Diego, CA, USA). The significant differences are indicated by (*) for (*p* < 0.05) and (**) for (*p* < 0.01) compared to the control.

## 3. Results

### 3.1. Monocytes Showed Differentiation into Macrophages by Co-Culture with the Supernatants of EBV-Positive NK-Cells

**Figure 1:**
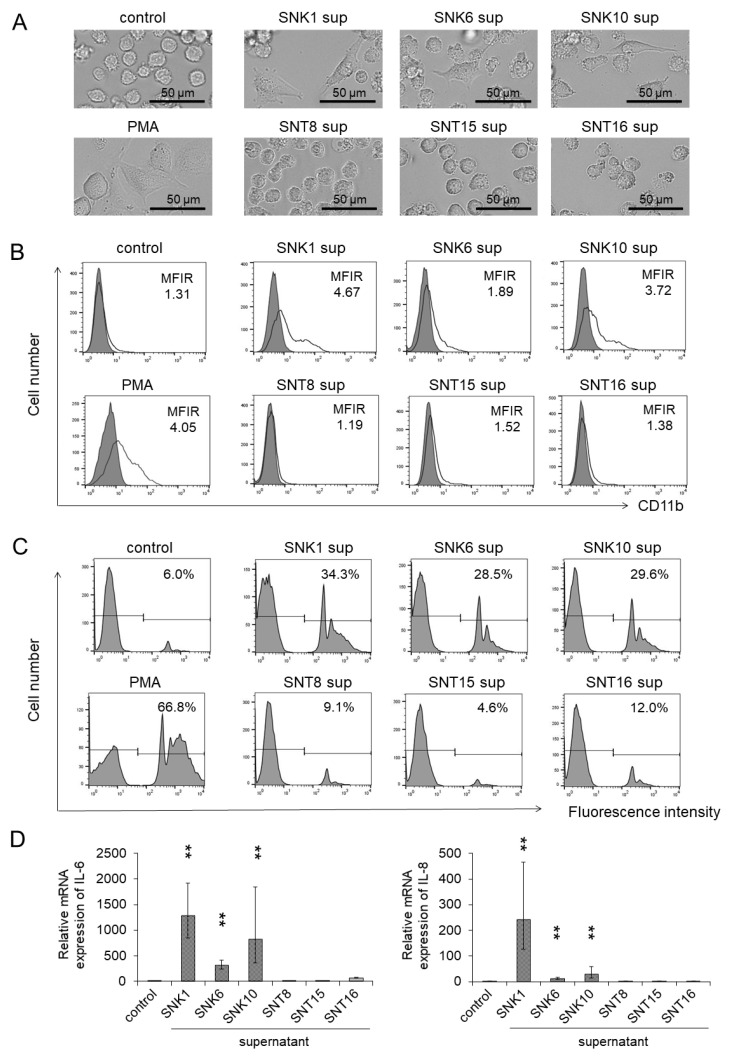
Macrophage-like differentiation and activation were promoted by the supernatants of EBV-positive NK-cell lines to THP-1 cells. THP-1 cells were co-cultured with each supernatant of EBV-positive T- or NK-cell lines for 72 h. After the co-culture, the target cells were subjected to each assay. The same amount of culture media of EBV-positive T- or NK-cell lines was added as an untreated control. PMA, a cell differentiation regulator, was used as a positive control. (**A**) The representative morphology of THP-1 cells was photographed by an optical microscope. (**B**) The CD11b expression on THP-1 cells was determined by flow cytometry using an antibody to CD11b (open histogram) or isotype-matched control (gray, shaded histogram). The mean fluorescent intensity of CD11b was normalized by the mean of isotype-matched control and is shown as the MFIR (mean fluorescence intensity rate). (**C**) The phagocytosis ability of THP-1 cells was examined by a fluorescent beads uptake assay. After co-culturing with each supernatant, THP-1 cells were incubated with the fluorescent beads for 4 h, and the uptake of these beads was detected by flow cytometry. The rate of fluorescence intensity positive was estimated as phagocytosis ability. (**D**) The mRNA expression of *IL-6* and *IL-8* in THP-1 cells was analyzed by qRT-PCR assay. The expression was normalized to *GAPDH* mRNA. The data are shown as the mean ± SD (*n =* 3). Statistical analyses were assessed and indicated as ** *p* < 0.01 compared to the control.

First, we investigated if the products of EBV-positive T- or NK-cell lines had effects on monocytic cell lines. The supernatants of EBV-T-cell lines and EBV-NK-cell lines were used as stimulators. As shown in Figure 1A, co-culturing with the supernatants of EBV-NK-cell lines induced macrophage-like morphological change of THP-1 cells: they enlarged, flattened, and adhered to the bottom of culture flasks. The supernatants of EBV-T-cell lines did not show such phenomena. We then examined the expression of CD11b, which is a cell surface marker of a macrophage. Co-culturing with the supernatants of EBV-NK-cell lines induced CD11b expression on the surface of THP-1 cells, although co-culturing with EBV-T-cell lines did not (Figure 1B). We also examined the phagocytosis ability using fluorescence microspheres. The uptake of microspheres increased by co-culturing with the supernatants of EBV-NK-cell lines remarkably in comparison to the supernatants of EBV-T-cell lines (Figure 1C). As shown in Figure 1D, the expressions of mRNA of macrophage-derived inflammatory cytokines, *IL-6* and *IL-8,* were enhanced by co-culturing with the supernatants of EBV-NK-cell lines. Contrarily, the expressions were hardly enhanced by the co-culturing with the supernatants of EBV-T-cell lines. These results indicate that the supernatants of EBV-positive NK-cells induced the differentiation of THP-1 cells into functional macrophages.

**Figure 2:**
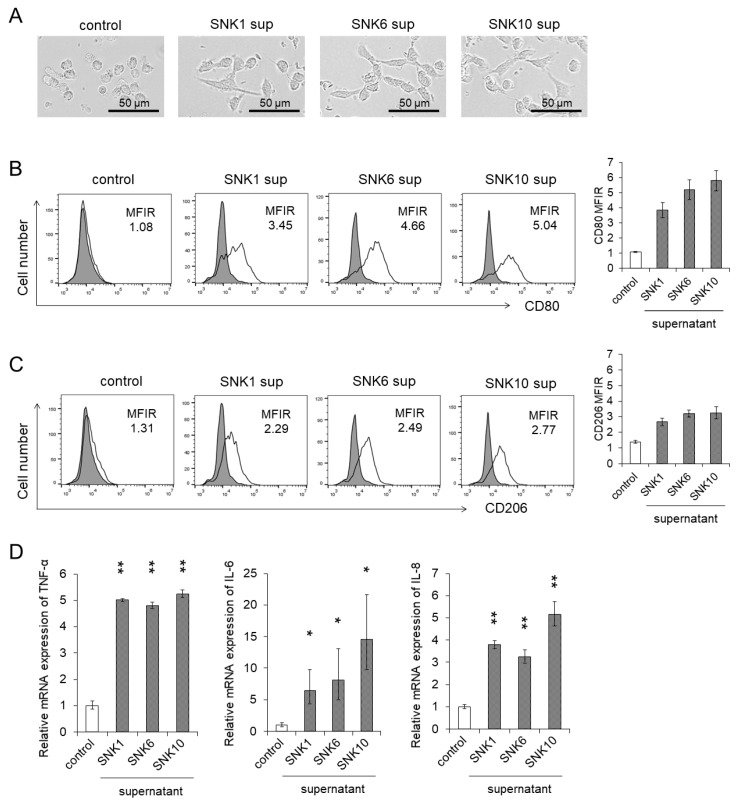
The differentiation of human monocytes to macrophages and activation were promoted by the supernatants of EBV-positive NK-cell lines. Human monocytes were co-cultured with each supernatant of EBV-positive NK-cell lines for 24 h. The same amount of culture media of EBV-positive NK-cell lines was added as an untreated control. After the co-culture, the target cells were subjected to each assay. (**A**) A representative morphology of human monocytes was pictured using an optical microscope. (**B**,**C**) The CD80 and CD206 expression on human monocytes was determined by flow cytometry. The representative figure is shown on the left panel indicating CD80 and CD206 (open histogram) or isotype-matched control (gray, shaded histogram). The mean fluorescent intensity of CD80 and CD206 was normalized by that of isotype-matched control and expressed as MFIR. (**D**) The mRNA expression of *TNF-α*, *IL-6* and *IL-8* in human monocytes was analyzed by qRT-PCR assay. The expression was normalized to *GAPDH* mRNA. The data are shown as the mean ± SD of three independent healthy donors (*n =* 3). Significant differences are indicated as * *p* < 0.05 and ** *p* < 0.01 compared to the control.

We validated the results using primary human monocytes derived from healthy donors. We isolated monocytes from the peripheral blood of healthy donors as described in the Materials and Methods. We confirmed that 86.5% of the isolated cells were positive for CD14, which is a surface marker of human monocytes (Figure S1). As shown in Figure 2A, these monocytes showed morphological changes when co-cultured with the supernatants of EBV-NK-cell lines as THP-1 cells did. After the co-culture, the surface expressions of CD80, a M1 macrophage marker, and CD206, a M2 macrophage marker, were upregulated on monocytes (Figure 2B,C). Furthermore, the mRNA of *TNF-α*, *IL-6,* and *IL-8,* which are produced by M1 and M2 macrophages, increased in human monocytes after the co-culture (Figure 2D). These results suggest that some humoral factors, such as cytokines secreted from EBV-positive NK cells, may possibly induce the differentiation into M1- and M2-like macrophages from monocytes and their activation.

### 3.2. Co-Culture with the Supernatants of EBV-Positive NK-Cells Promoted Procoagulant Activity of Monocytes through the Upregulation of TF Expression

**Figure 3:**
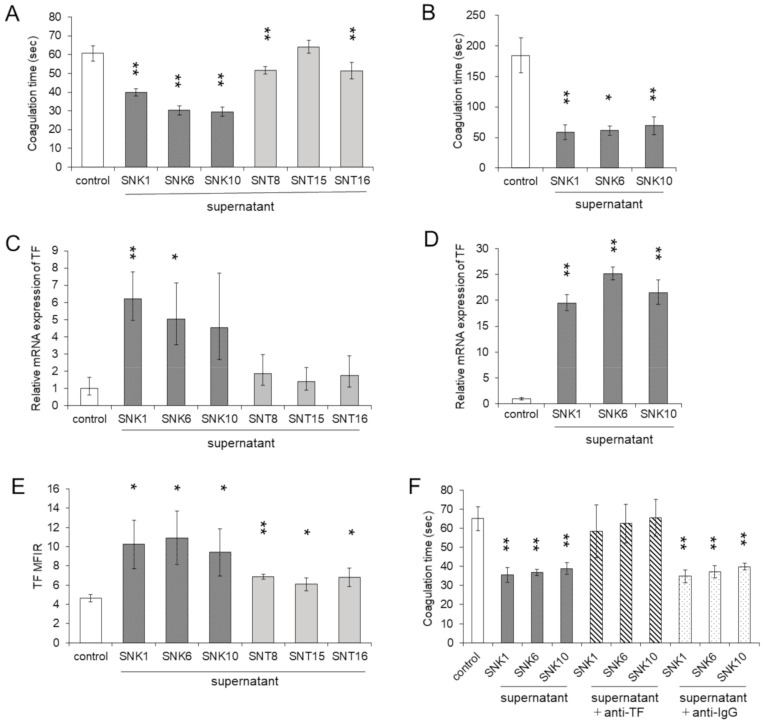
Procoagulant activity was enhanced by the supernatants of EBV-positive NK-cell lines. THP-1 cells and human monocytes were co-cultured with each supernatant of EBV-positive T- or NK- cell lines for 24 h. The same amount of culture media of EBV-positive T- or NK-cell lines was added as an untreated control. After the co-culture, the target cells were subjected to each assay. (**A**) The procoagulant activity (PCA) on THP-1 cells was measured by normal plasma-based recalcification time (*n =* 6). The shortening of coagulation time indicates increased the PCA. (**B**) The PCA on human monocytes of three independent healthy donors was measured (*n =* 3). (**C**) The mRNA expression of the *tissue factor (TF)* in THP-1 cells was examined by qRT-PCR (*n =* 3). The expression was normalized to *GAPDH* mRNA. (**D**) The mRNA expression of *TF* in human monocytes of three independent healthy donors was measured (*n =* 3). (**E**) The cell surface TF antigen on THP-1 cells was analyzed by flow cytometry using an antibody to TF or isotype-matched control. The mean fluorescent intensity of TF was normalized by that of isotype-matched control and expressed as MFIR (*n =* 4). (**F**) To investigate the effects of cell surface TF on PCA, THP-1 cells were treated with mouse monoclonal anti-human TF antibody or the same amount of irrelevant IgG. After the treatment of antibody, the cell surface PCA was assessed (*n =* 4). The data are shown as the mean ± SD. Statistical analyses were assessed and are indicated as * *p* < 0.05 and ** *p* < 0.01 compared to the control.

DIC is a major complication of HLH. Activated macrophages enhance the expression of molecules that promote blood coagulation, such as TF and PS, on their surface. The expression of these molecules will eventually cause DIC [16]. We examined if the culture supernatants of EBV-positive T- or NK-cell lines could cause an increase of cell surface PCA on monocytes as described in the Materials and Methods. PCA was significantly activated in THP-1 cells treated with the supernatants of EBV-positive NK-cell lines, whereas the effects of the supernatants from EBV-positive T-cell lines were minimal (Figure 3A).

We validated the findings with human monocytes. PCA was significantly activated in monocytes treated with the supernatants of EBV-NK-cell lines (Figure 3B). We investigated the expression of TF, which can activate PCA in THP-1 cells and monocytes. After stimulating with each supernatant of EBV-NK-cell lines, the mRNA expression of *TF* was enhanced in THP-1 cells (Figure 3C). The mRNA expression of *TF* in human monocytes was also enhanced by the supernatants of EBV-NK-cell lines (Figure 3D). The cell surface expression of TF protein was also significantly enhanced in THP-1 cells (Figure 3E).

The next step was to examine if TF directly enhanced PCA in the cells incubated with the supernatant of EBV-NK-cell lines. In THP-1 cells, the upregulated cell surface PCA was blocked by co-culturing with the anti-TF antibody (Figure 3F). We also examined the effects of the coagulation protein PS, which induces blood coagulation by the facilitating factor Xa and thrombin formation [17]. More expressions of PS were induced by the supernatants of EBV-NK-cell lines compared to the control (Figure S2A). However, enhanced PCA by the supernatants of EBV-NK-cell lines was not influenced by Annexin V, an antagonist of PS (Figure S2B). These results suggest that PCA increased by the supernatants of EBV-NK-cell lines was induced by the induction of TF.

### 3.3. Inflammatory Cytokines in the Supernatants of Cultured EBV-Positive NK-Cell Neoplasms

**Figure 4:**
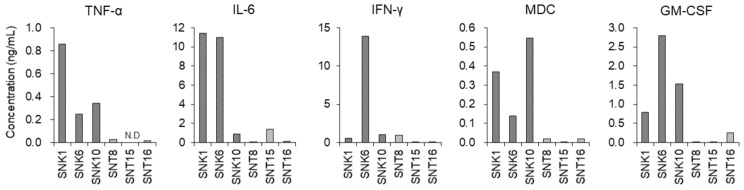
Identification of cytokines in the supernatants of EBV-positive T- or NK-cell lines. The cytokine concentrations in the supernatants of EBV-positive T- or NK-cell lines were examined using a cytokine multiplex assay. The concentrations of TNF-α, IL-6, IFN-γ, MDC, and GM-CSF are shown. N.D.; not detected.

These results indicate that some humoral factors, which are produced and secreted by EBV-NK-cells, may be contributing to the activation of PCA and the differentiation to macrophages of monocytes. To prove our hypothesis, we investigated which humoral factors influenced the effects on monocytes. Since the levels of inflammatory cytokines increased in the plasma of EBV-positive T- or NK-cell neoplasms [11,12,18], we performed cytokine multiplex analysis to identify the responsible cytokines for the differentiation and the activation of monocytes (Figure S3).We detected five proinflammatory cytokines, TNF-α, IL-6, IFN-γ, MDC, and GM-CSF, which showed remarkably high levels in the supernatants of EBV-NK-cell lines compared to those in the supernatants of EBV-T-cell lines (Figure 4).

There are reports stating that the concentrations of TNF-α, IL-6 and IFN-γ were elevated in the blood of EBV-positive NK-cell neoplasms [19,20,21]. We measured the concentration in the blood obtained from four patients of EBV-positive NK-cell neoplasms with the complication of HLH. Among the five cytokines, the IFN-γ concentration was remarkably high and at a similar level as in the supernatants (Table 1). We investigated the concentration of IFN-γ in the culture supernatants of human NK cells of two healthy donors and two types of EBV-positive NK-cell lines, SNK1 and SNK6 cells, in three different concentrations. As shown in Figure S4, the culture supernatants of EBV-positive NK-cell lines contained much more IFN-γ than the supernatants of human NK cells in the same concentration. From this finding, we assumed that EBV-positive NK-cells produce more IFN-γ compared to human NK cells. In patient blood, GM-CSF was not detected, and MDC was in the normal range (Table 1). Based on these results, we decided to focus on IFN-γ.

### 3.4. IFN-γ Directly Enhanced the Differentiation and the Procoagulant Activity of Monocytes

**Figure 5:**
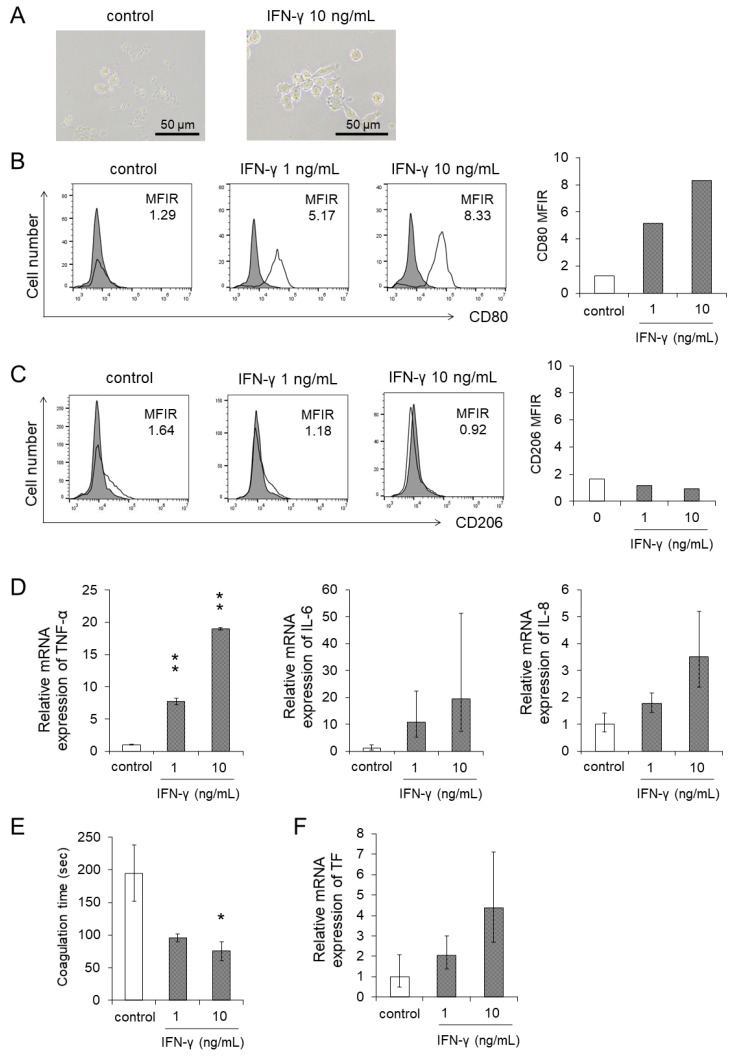
Differentiation and procoagulant activity were promoted by the stimulation of IFN-γ. Human monocytes were treated with recombinant IFN-γ for 24 h. The same amount of culture media of EBV-positive T- or NK-cell lines was added as an untreated control. After the treatment, the target cells were subjected to each assay. (**A**) The representative morphology was pictured using an optical microscope. (**B**,**C**) The CD80 (**B**) and CD206 (**C**) expression on human monocytes was determined by flow cytometry. The representative figure is shown on the left panel indicating CD80 and CD206 (open histogram) or isotype-matched control (gray, shaded histogram). The mean fluorescent intensity of CD80 and CD206 was normalized by that of isotype-matched control and expressed as MFIR. (**D**,**F**) The mRNA expression of *TNF-α*, *IL-6*, *IL-8* (**D**), and *TF* (**F**) in human monocytes was analyzed by qRT-PCR assay (*n =* 3). The expression was normalized to *GAPDH* mRNA. (**E**) The PCA on human monocytes was measured using the normal plasma-based recalcification time (*n =* 3). Shortening of the coagulation time indicates increased PCA. The data are shown as the mean ± SD of three independent healthy donors. Statistical analyses are assessed and indicated as * *p* < 0.05 and ** *p* < 0.01 compared to the control.

Finally, we investigated the direct effects of IFN-γ in the differentiation to macrophages and the upregulation of PCA of monocytes. We stimulated monocytes with the recombinant IFN-γ in different concentrations as shown in Figure 5. A macrophage-like morphological change of monocytes was observed by the IFN-γ treatment (Figure 5A). IFN-γ directly induced CD80 expression of monocytes (Figure 5B). However, CD206, which is an M2 macrophage marker, was not induced by IFN-γ (Figure 5C). We examined the effects of IFN-γ on the expression of the inflammatory cytokines. The production of mRNA of *TNF-α*, *IL-6,* and *IL-8,* which are produced by M1 and M2 macrophages, increased after the stimulation by IFN-γ in human monocytes (Figure 5D). Based on these results, we concluded that IFN-γ induced the differentiation of monocytes to M1 macrophages. After the stimulation of IFN-γ, PCA (Figure 5E) and *TF* expression (Figure 5F) were enhanced in monocytes although we could not observe a statistical significance due to the large variability.

We also investigated the effects of TNF-α and IL-6. Their concentrations were notably higher in the supernatants of EBV-NK-cell lines compared to those of EBV-T-cell lines, and they were also high in the patients’ blood (Table 1). The morphological changes and the induction of CD80 and CD206 expression were not observed in either TNF-α- or in IL-6-treated monocytes (Figure S5A–C). IL-6 did not induce the activation of PCA with the upregulation of *TF* and the mRNA expression of *TNF-α, IL-6*, and *IL-8* of monocytes. TNF-α induced the activation of PCA and only *IL-8* mRNA in monocytes (Figure S5D–F). These results suggest that IFN-γ plays a critical role to differentiate and to activate M1 macrophages in EBV-positive NK-cell neoplasms leading to HLH.

## 4. Discussion

In this study, we demonstrated that the differentiation of monocytes to M1- and M2-like macrophages was induced by co-culturing with the supernatants of EBV-positive NK cells. The co-culture also enhanced the procoagulant activity of monocytes by the upregulating TF expression. We examined the cytokines in the supernatants under the assumption that the products from EBV-positive T or NK cells were the causes of differentiation and activation. We determined that IFN-γ, which abundantly existed not only in the supernatants of the EBV-NK-cell lines but also in the blood of EBV-positive NK-cell neoplasms patients with HLH, contributed to M1 macrophage differentiation and the upregulation of the procoagulant activity of monocytes.

It has been reported that a large number of EBV-infected tumor cells exist in the peripheral blood of EBV-positive NK-cell neoplasms particularly of ANKL and CAEBV with the elevation of IFN-γ [12,18,19,20,21]. There are also reports of EBV-positive NK cells directly producing IFN-γ [22,23]. M1 macrophages produce inflammatory cytokines and are possible cause of HLH. These findings suggest that IFN-γ produced from the tumor cells in EBV-positive NK-cell neoplasms plays a role in the activation of M1 macrophages and HLH development.

The macrophages differentiated from the monocytes co-cultured with EBV-positive NK-cell supernatants expressed the surface markers of not only M1 but also M2 macrophages. Unlike M1 macrophages, M2 macrophages negatively regulate immune reaction and function as tumor-associated macrophages (TAM) enhancing the survival of tumor cells. In this study, IFN-γ did not induce the expression of CD206, a M2 macrophage marker. Which factor contributes to the M2 macrophage-like differentiation of monocytes? Recently, cell-free RNAs secreted from EBV-positive tumor cells are gaining attention. It was reported that EBV-derived miRNAs were detected in the peripheral blood of EBV-positive neoplasms [24,25,26]. Interestingly, Higuchi et al. reported that exosomes secreted from EBV-positive B cell neoplasms contained EBV-derived miRNAs [27]. They also detected that miRNAs induced the immune regulatory phenotype of TAM. Thus, EBV-derived miRNAs may have roles in the development of HLH in EBV-positive T- or NK-cell neoplasms by inducing the differentiation of M2 macrophages. Further study is necessary to prove the hypothesis and is ongoing in our laboratory.

Several study groups, including us, have reported that STAT3 is constitutively activated in EBV-positive NK-cell neoplasms [22,28]. In addition, Küçük et al. reported the presence of activating mutations in the Src homology 2 domains of *STAT3* in EBV-positive NK-cell lines and in ENKL patient cells [29]. STAT3 is a transcriptional factor and upregulates the production of IFN-γ [30]. Therefore, activated STAT3-induced production of IFN-γ may be one of the causes of high concentration of IFN-γ in the supernatants of cultured EBV-positive NK-cell lines. However, as we previously reported, the constitutive activation of STAT3 was observed not only in EBV-positive NK cells but also in EBV-positive T cells [22]. In the supernatants of SNT15 and SNT16, IFN-γ was undetectable. Why was IFN-γ protein not produced in these cells despite the constitutive activation of STAT3? The production may depend on the characteristics of each cell. SNT15 is a γδ T-cell line, and γδ T cells may not produce IFN-γ [31]. EBV-positive T-cell neoplasms also consist of γδ T cells and αβ T cells. We need further extensive studies using clinical samples to confirm whether IFN-γ production is limited to EBV-positive NK cells or not.

EBV-positive neoplasms are chemoresistant. In particular, ANKL and CAEBV with HLH have high mortality. It is crucial to regulate HLH and DIC in bridging to hematopoietic stem cell transplantation (HSCT), a curative treatment strategy of CAEBV. In this study, we suggest that IFN-γ is an attractive therapeutic target for EBV-positive NK-cell neoplasms accompanied by HLH. According to our previous report, ruxolitinib, a JAK1/2 inhibitor suppressed STAT3 activation and IFN-γ production of EBV-positive NK cells in patients’ cells [22]. Recently, we also observed that a proteasome inhibitor bortezomib suppressed IFN-γ production not only in EBV-positive T- or NK-cells but also in xenograft model of CAEBV [32]. Additionally, emapalumab, a neutralizing anti-IFN-γ monoclonal antibody has been reported for its efficacy to treat primary HLH [33]. These reagents can be the candidates of novel medicine to prevent and to treat HLH in EBV-positive NK-cell neoplasms. We highly anticipate the development of treatment strategy targeting IFN-γ.

## 5. Conclusions

IFN-γ produced by EBV-positive neoplastic NK-cells is responsible for inducing the differentiation and the activation of macrophages. Suppressing IFN-γ may regulate HLH in EBV-positive NK-cell neoplasms.

## Figures and Tables

**Table 1:** The concentration of the inflammatory cytokines in the blood of the EBV-positive NK-cell neoplasms accompanied by HLH.

Case Number	Age	Gender	Disease	TNF-α (pg/mL)	IL-6 (pg/mL)	IFN-γ (pg/mL)	MDC (pg/mL)	GM-CSF (pg/mL)	Specimen
1	21	F	ANKL	<5.5	3.7	4255.0	70.1	N.D.	plasma
2	35	F	CAEBV (CD56 type)	30.8	110.6	571.7	157.2	N.D.	plasma
3	36	M	ENKL	12.0	23.1	2863.3	155.0	N.D.	plasma
4	35	F	CAEBV (CD56 type)	<5.5	12.0	11,608.0	44.0	N.D.	serum

F; female, M; male, EBV; Epstein–Barr virus, ANKL; aggressive NK-cell leukemia, CAEBV; chronic active Epstein–Barr virus infection, ENKL; extranodal NK/T-cell lymphoma, nasal type, N.D.; not detected.

## Data Availability

Data is contained within the article or Supplementary Material.

## Supplementary Materials

The following are available online at https://www.mdpi.com/article/10.3390/cancers13205097/s1, Figure S1: Enrichment of human monocytes, Figure S2: Induced phosphatidylserine was not related to the procoagulant activity, Figure S3: Identification of cytokines in the supernatants of EBV-positive T- or NK-cell lines, Figure S4: The concentration of IFN-γ in the supernatant of healthy donors’ NK cells and EBV-positive NK-cell lines, Figure S5: Effects of TNF-α, IL-6 and IFN-γ on the differentiation and procoagulant activity of human monocytes.
