# Supplementary material for: Interferon-γ Produced by EBV-Positive Neoplastic NK-Cells Induces Differentiation into Macrophages and Procoagulant Activity of Monocytes, Which Leads to HLH
Source: Cancers (Basel). 2021 Oct 12;13(20):5097. doi: 10.3390/cancers13205097 (PMC8533691; doi:10.3390/cancers13205097)
Supplement: Supplementary file 1 [file cancers-13-05097-s001.zip › cancers-1398776-supplementary.pdf]

Supplementary Materials

Interferon-γ Produced by EBV-Positive Neoplastic NK-Cells Induces Differentiation into Macrophages and Procoagulant Activity of Monocytes, Which Leads to HLH

Mayumi Yoshimori, Miwako Nishio, Ayaka Ohashi, Megumi Tateishi, Ayaka Mimura, Naomi Wada, Minori Saito, Norio Shimizu, Ken-Ichi Imadome and Ayako Arai

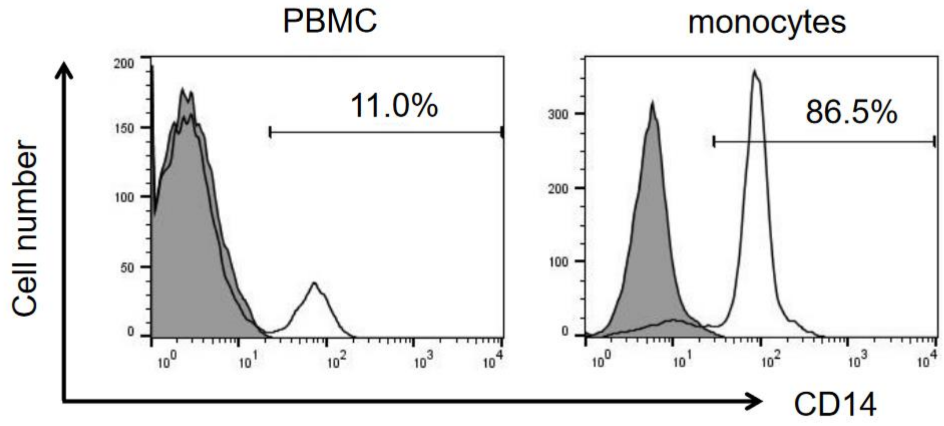

Figure S1. Enrichment of human monocytes. Human monocytes were isolated using an indirect magnetic labeling system from the PBMC. The CD14-positive monocytes which were 11.0% in the PBMC were enriched by 86.5% using the magnetic system.

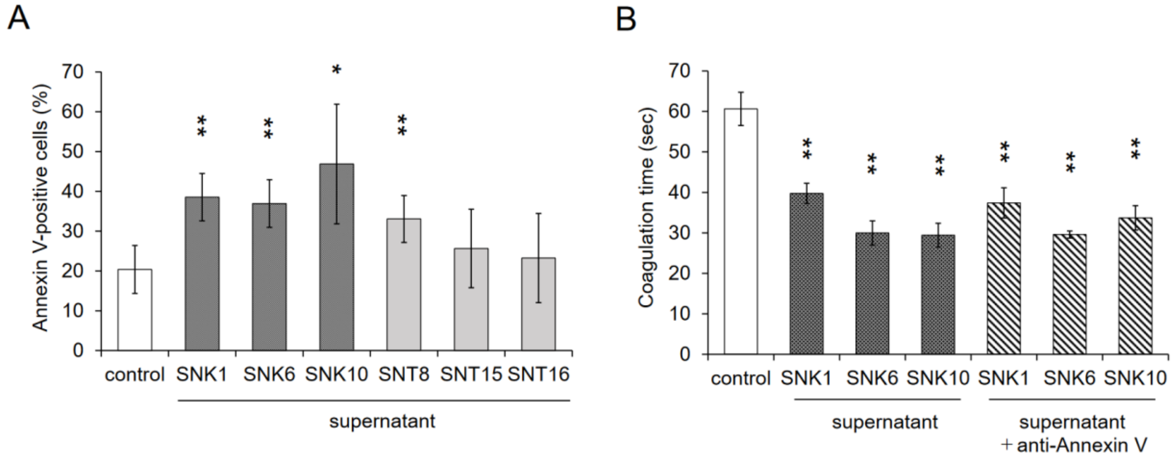

Figure S2. Induced phosphatidylserine was not related to the procoagulant activity.THP-1 cells were co-cultured with each supernatant of EBV-positive T- or NK- cell lines for 24 h. After the co-culture, the cells were subjected to each assay. (**A**) Cell surface phosphatidylserine (PS) antigen on THP-1 cells was analyzed by flow cytometry using an Annexin V antibody. The rate of Annexin Vpositive cells was shown as mean ± SD ($n = 4$). (**B**) To investigate the effects of cell surface PS on PCA, THP-1 cells were treated with Annexin V antibody. After the treatment of antibody, cell surface PCA was assessed. The data are shown mean ± SD ($n = 4$). Significant differences are indicated as * $p < 0.05$ and ** $p < 0.01$ comparing to the control.

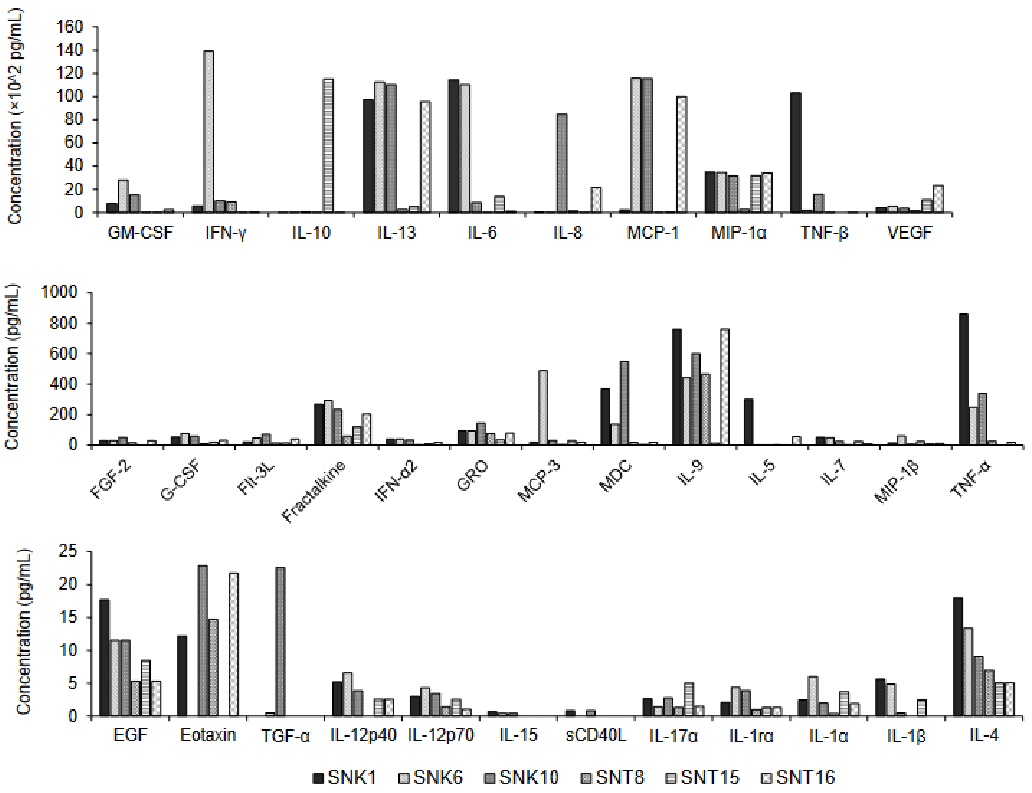

Figure S3. Identification of cytokines in the supernatants of EBV-positive T- or NK-cell lines. The cytokine concentrations in the supernatants of EBV-positive T- or NK-cell lines were examined using cytokine multiplex assay.

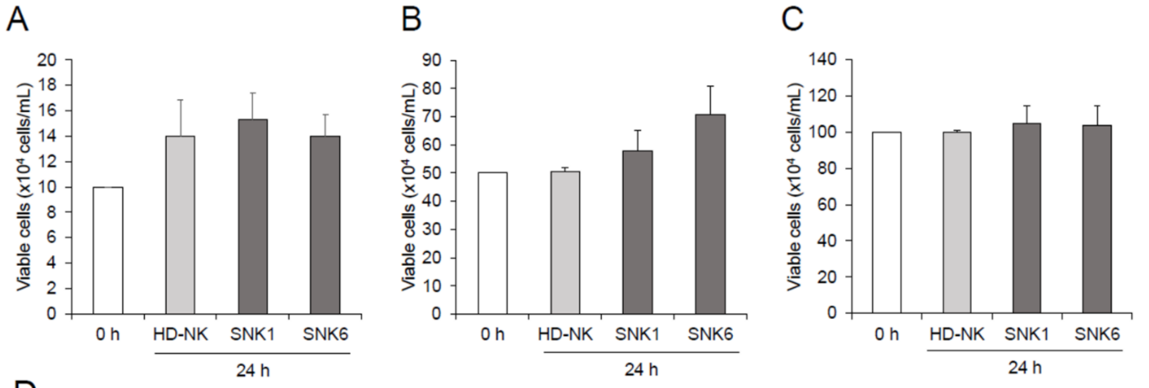

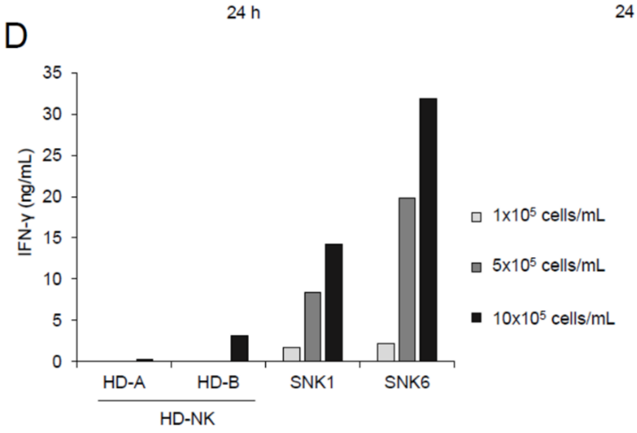

IFN-γ concentration in the supernatants of each cells (ng/mL)

	HD-A	HD-B	SNK1	SNK6
1 x 10^5 cells/mL	N.D.	N.D.	1.64	2.20
5 x 10^5 cells/mL	N.D.	0.07	8.37	19.84
10 x 10^5 cells/mL	0.30	3.10	14.17	31.84

Figure S4. The concentration of IFN-γ in the supernatant of healthy donors' NK cells and EBV-positive NK-cell lines. NK cells (HD-NK: HD-A and HD-B) of two healthy donors and EBV-positive NK-cell lines, SNK1 and SNK6, were cultured for 24 h and the supernatants were collected. We examined the proliferation of the cells in three different concentrations of 1 × 10^5 cells/mL (**A**), 5 × 10^5 cells/mL (**B**), and 10 × 10^5 cells/mL (**C**). The concentration of IFN-γ in each supernatant was measured (**D**). N.D.; not detected.

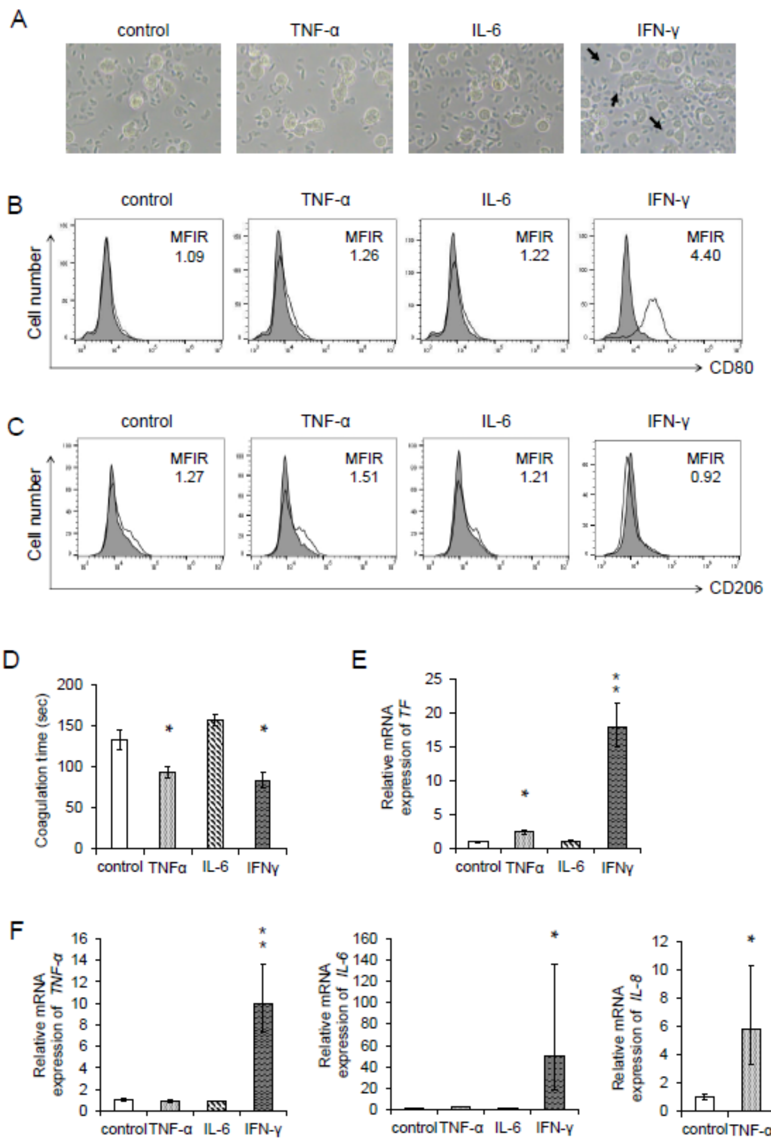

Figure S5. Effects of TNF-α, IL-6 and IFN-γ on the differentiation and procoagulant activity of human monocytes. Human monocytes were treated with 1.0 ng/mL of TNF-α, 20 ng/mL of IL-6, and 10 ng/mL of IFN-γ for 24 h. After the treatment, the target cells were subjected to each assay. (**A**) Representative morphology was pictured by an optical microscope. (**B**,**C**) The CD80 (B) and CD206 (C) expression on human monocytes was determined by flow cytometry. The representative figure is shown on the left panel indicating CD80 (open histogram) or isotype-matched control (gray, shaded histogram). The mean fluorescent intensity of CD80 was normalized by that of isotype-matched control and expressed as MFIR. (**D**) PCA was measured by normal plasma-based recalcification time. Shortening of the coagulation time indicates increased PCA. (**E**,**F**) The mRNA expression of TF, TNF-α, IL-6 and IL-8 was analyzed by qRT-PCR assay. The expression was normalized to *GAPDH* mRNA. The data are shown as mean ± SD of three independent healthy donors (n = 3). Significant differences are indicated as * $p < 0.05$ and ** $p < 0.01$ comparing to the control.

© 2021 by the authors. Licensee MDPI, Basel, Switzerland. This article is an open access article distributed under the terms and conditions of the Creative Commons Attribution (CC BY) license (http://creativecommons.org/licenses/by/4.0/).
